# Smartphone ophthalmoscopy: patient and student practitioner perceptions

**DOI:** 10.1007/s10916-019-1477-0

**Published:** 2019-12-03

**Authors:** Manbir Nagra, Byki Huntjens

**Affiliations:** 1 School of Health Sciences and Social Work, University of Portsmouth, St Michael’s Building, Portsmouth, PO1 2DT UK; 2 Division of Optometry and Visual Science, Centre for Applied Vision Research, City, University of London, Northampton Square, London, EC1V 0HB UK

**Keywords:** Ophthalmoscopy, Optometry, Smartphone, Technology, Telehealth, User engagement, Medical education

## Abstract

It can take several years to become proficient at direct ophthalmoscopy; the instrument’s single eyepiece allows only one individual to view the image at a time, which is considered disadvantageous during teaching. The introduction of smartphone ophthalmoscopes enables groups of teachers and students to view images together which could encourage peer-to-peer learning. In addition, the technology is significantly cheaper than the direct ophthalmoscope. User acceptability and engagement is essential to the success of any (medical) technological innovation. We sought to understand student opinions of a new commercially-available smartphone device for fundus examination, and compare usability to the traditional ophthalmoscope, from the perspective of both student practitioners and patients. Fifty-four undergraduate optometry students with prior experience of the traditional direct ophthalmoscope were asked to examine at least one eye with the D-EYE smartphone ophthalmoscope and also given an opportunity to experience the D-EYE from a patient’s perspective. Minimal instructions were provided and all examinations conducted through undilated pupils. Participants completed an opinion survey to feedback on aspects such as the ease of handling and working distance. Compared to the direct ophthalmoscope, 92% of students preferred the (longer) working distance of the D-EYE; 77% felt it was easier to handle; and 92% preferred the patient experience with the D-EYE. Despite the positive feedback, only 43% of students preferred the D-EYE when assuming the role of the practitioner. Free text responses indicated that students felt the D-EYE may be most useful as a teaching tool. Student opinions indicated that smartphone ophthalmoscopes are an effective training tool for students as an accompaniment to learning the traditional ophthalmoscope method.

## Introduction

Direct ophthalmoscopy forms an integral aspect of the optometrist and medic’s armamentarium in ophthalmological and neuro-ophthalmological assessment. Direct ophthalmoscopy allows for rapid inspection of the internal oculus; often without the need for pupillary dilation. The technique does, however, suffer from a number of disadvantages including a very short working distance; limited field of view; and, when examining highly ametropic eyes, the resultant image is subject to significant difference in perceived image size.

It can take several years to become proficient at direct ophthalmoscopy; the instrument’s single eyepiece allows only one individual to view the image at a time, which can be particularly disadvantageous during teaching. Educators have trialled numerous approaches for teaching and assessment of direct ophthalmoscopy skills [1–8], including use of dummy eyes and simulators [1, 3–7], specially designed teaching ophthalmoscopes [2], and peer-to-peer learning games [8]. However, as far as we are aware in our capacity as educators, none of the aforementioned teaching methods have been widely adopted.

Reports indicate medics often lack confidence in their direct ophthalmoscopy skills [9, 10], and feel more training is required [11]. Others have, however, attributed a lack of ophthalmoscopic investigation on a deficit of available equipment [12]. As may be expected, individuals who seldom use their direct ophthalmoscopes risk the diminishment of their skills [13, 14].

Alternative methods of imaging the fundus, such as photography, have shown to be comparable and in some cases superior to direct ophthalmoscopy in the detection of retinal disease [15, 16]. Further, there appears to be a preference by some students for use of fundus photography over direct ophthalmoscopy [17]. Use of technologies such as fundus cameras also opens up the possibility of sharing the task of obtaining fundus photographs with other (assisting) members of the patient care team [16].

The recent introduction of smartphone ophthalmoscopes [18, 19] is perhaps a predictable development given the current trend of smartphone based healthcare devices and apps. In ophthalmology, smartphone apps have been developed for use in patient education, as patient assessment tools (e.g. vision testing), reference databases for clinicians (e.g. pathology grading systems) and for use within telehealth [ 20]. While there is evidence supporting use of smartphones in various areas of ophthalmology [20–23]; evidence of their usefulness as an educational tool is still emerging. Early reports suggest medical students view smartphone ophthalmoscopes favourably and are more likely to make correct and faster diagnoses than when using direct ophthalmoscopes [24, 25]. From a pedagogical perspective, the use of technologies with which students are already familiar, i.e. smartphones, may help to increase student engagement and enhance the learning experience. A more overt pedagogical advantage of the smartphone ophthalmoscope is the potential for groups of teachers and students to view fundus images together, hence facilitating peer-to-peer learning.

The purpose of this study is to understand student opinions of a commercially available smartphone device for fundus examination, the D-EYE, and to compare its usability to the traditional ophthalmoscope, from both the perspectives of an optometry student practitioner and patient.

## Methods

Fifty-four undergraduate optometry students, with prior experience of the traditional direct ophthalmoscope, were recruited during 2017 to evaluate the D-EYE smartphone ophthalmoscope attachment (D-EYE Srl, Padova, Italy). The study received ethics approval via the Optometry departmental ethics committee and all participants provided written informed consent prior to taking part. All aspects of the study conformed to the tenets of the Declaration of Helsinki.

The D-EYE is a commercially available app-enabled device which attaches to specific models of smartphones via an internal magnet and a customised bumper. The device enables real-time retinal imaging and allows both photography and video recording of the retinal image. Field of view is approximately 20° at a distance of 1 cm from the patient’s eye for a dilated pupil, Russo et al. (2015) [19] advise that image quality may be reduced for pupil sizes of less than 2.5 mm.

Rather than the practitioner focusing the machine manually, as is the case with the traditional direct ophthalmoscope, the device utilises the smartphone’s internal autofocus function which enables compensation for refractive errors of approximately −12 to +6D [19].

Participants were asked to examine at least one eye with the D-EYE smartphone attachment and an iPhone 5 (Apple Inc., Cupertino, CA, USA), and were given an opportunity to assume the role of the patient. Minimal instructions were provided and all examinations were conducted through undilated pupils. Participants completed an opinion survey to feedback on aspects such as the ease of handling, working distance, and overall preference in comparison to traditional ophthalmoscopy from the perspective of both the practitioner and the patient (see Table 1). The opinion survey used a combination of Likert scale-based responses; free text responses; and binary (D-EYE or Direct ophthalmoscope) responses. The opinion survey was designed specifically for the study.

**Table 1:**
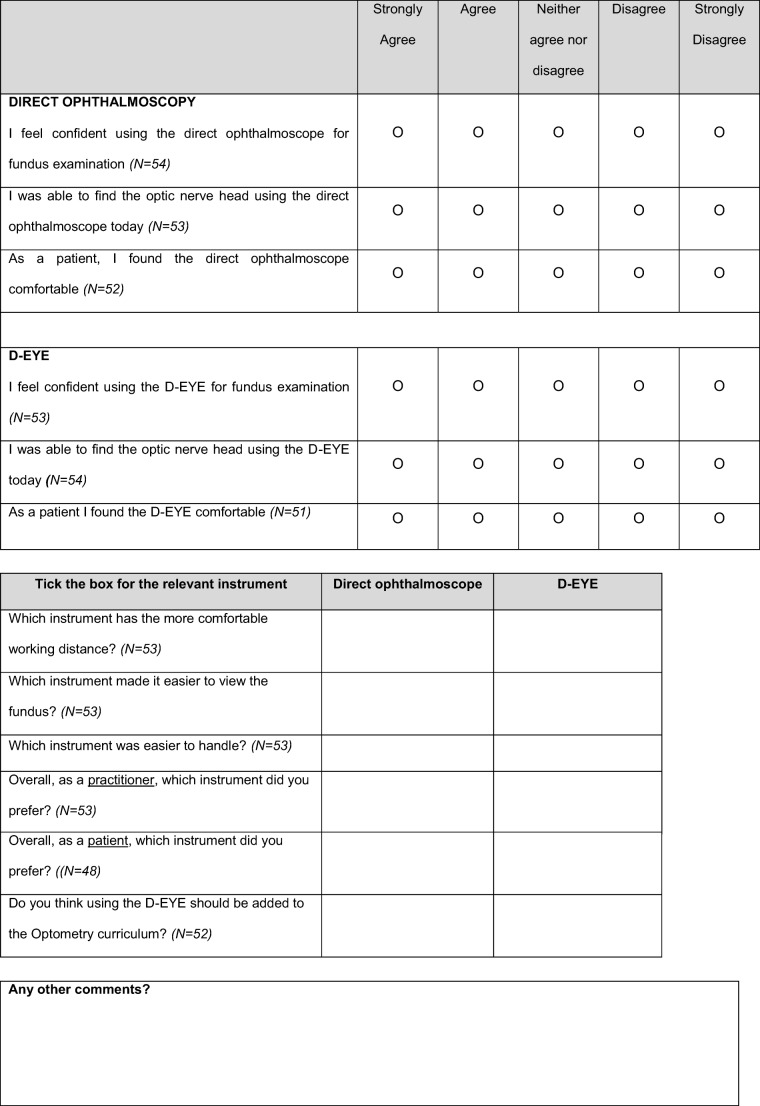
Opinion survey questions used to record participant responses following use of the D-EYE smartphone ophthalmoscope. Number of respondents to each section are also included to indicate cohort size

## Results

**Fig. 1:**
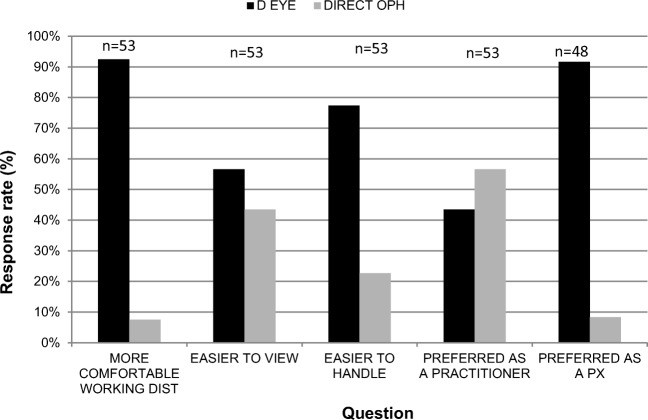
Indicates percentage of participants who agreed with the binary choice statements in relation to working distance; ease of view; ease of handling; and from the perspectives of practitioners and patients using D-EYE or direct ophthalmoscopy. Number of respondents for each section are shown

Fifty-four final-year optometry students took part in the study; each assumed the role of the practitioner and the role of the patient. Numbers of responses received to each of the questions are listed in Fig. 1. Percentages were calculated without the inclusion of non-respondents.

**Fig. 2:**
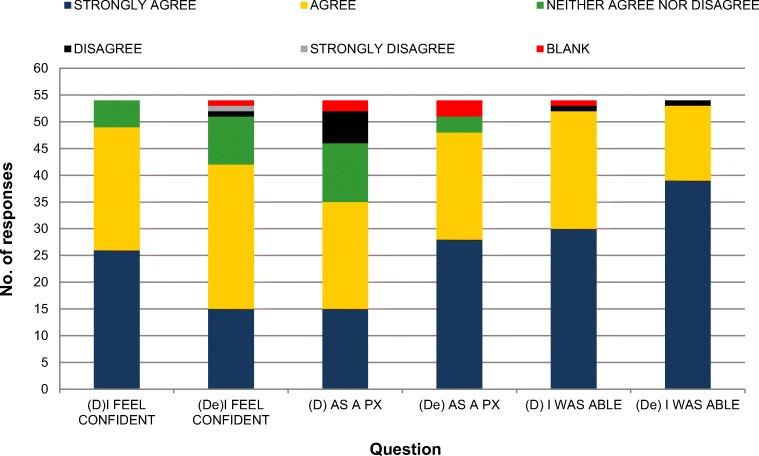
Number of responses to opinion survey using a 5-point Likert scale, see Table 1 for full list of questions

The majority of students ‘agreed’ or ‘strongly agreed’ that they felt confident using the D-EYE (79.2%) and direct ophthalmoscope (90.7%); the difference in confidence between the two techniques most likely reflects the prior experience in using the direct ophthalmoscope acquired during their undergraduate degree. Nevertheless, a similar percentage of students ‘agreed’ or ‘strongly agreed’ that they were able to locate the optic nerve head using the D-EYE and the direct ophthalmoscope (Fig. 2).

Compared to the direct ophthalmoscope, binary responses showed 92% of students preferred the (longer) working distance of the D-EYE; 77% felt the device was easier to handle; and 57% felt it was easy to view the fundus. However, most marked differences were observed in the patient experience of using the D-EYE. Ninety-two percent of participants preferred the patient experience with the D-EYE compared to the direct, and when asked whether the participants found the two procedures comfortable, 67.3% ‘agreed’ or ‘strongly agreed’ that the direct ophthalmoscope was comfortable compared to 94.1% for the D-EYE. Despite the positive feedback, only 43% of students preferred the D-EYE to the direct ophthalmoscope when assuming the role of the practitioner. When asked whether the D-EYE should be added to the syllabus 40 out of 54 participants agreed, 10 disagreed, with the remaining participants either not answering the questions (*n* = 2) or not providing a clear response (n = 2).

Twenty-nine out of 54 students provided free text responses regarding the D-EYE, these were coded as either positive (*n* = 12), negative (*n* = 6), or both positive and negative/neither (*n* = 11). In general, free text responses indicated that students felt the D-EYE was useful as a teaching tool and easy to use, but they found the peripheral retina difficult to visualise through the undilated pupil. The proportion of students who were able to locate the optic nerve head was similar for both devices (98%). We chose not to ask students to comment specifically upon the retinal periphery as they typically would not be asked to do so during initial direct ophthalmoscopy lessons. In addition, the pupils were undilated which can make it difficult to observe beyond the central retina.

## Discussion

Student opinion of the D-EYE ophthalmoscope indicated the device is perceived favourably from the perspective of the patient, and students preferred the longer 20–60 cm (cm) working distance of the D-EYE compared to the traditional 1–3 cm when using a direct ophthalmoscope. Overall, however, students indicated that they preferred to use the traditional direct ophthalmoscope; this may in part be due to their relative inexperience using the D-EYE.

The peripheral retina and macula were particularly difficult to view with the D-EYE, this was also likely to be due to the relative inexperience of using the instrument and lack of pupil dilation. The decision not to dilate the pupils was made to draw direct comparisons with the traditional ophthalmoscope, a technique generally taught and clinically performed without the use of mydriatic eye drops. The manufacturers of the D-EYE do, however, advise the use of mydriatics prior to full fundus examination as opposed to sole viewing of the optic nerve head (http://www.d-eyecare.com/en_GB/howtouse). Following this advice, a previous study has shown that during dilated fundus examination, the D-EYE is able to detect fundus abnormalities with sensitivity and specificity of 72% and 97%, respectively, when compared to traditional direct ophthalmoscopy [24]. While compared to slit lamp biomicroscopy, clinical significant macular oedema was detected with a sensitivity and specificity 81% and 98% respectively using the D-EYE system [26].

Several studies have shown that medics are generally not confident using the direct ophthalmoscope and instead would prefer to use fundus photographs [11, 12, 17]. The lack of confidence and absence of available equipment may be why some medics fail to examine the retina [11, 12]. For those individuals, the smartphone-based ophthalmoscope may offer an acceptable alternative for viewing regions such as the optic nerve head. Since the instrument requires minimal training and allows capture of photographic and video images, there is also scope to consider training ophthalmic nurses, and even non-clinical support staff to acquire the images on behalf of the clinicians. We conducted our study with optometry, as opposed to medical, students who are expected to demonstrate competence with the direct ophthalmoscope throughout their undergraduate studies and beyond. Mamtora et al. (2018) showed medical students were more likely to make a correct diagnosis of an ophthalmological condition when using the D-EYE rather than the direct ophthalmoscope [24]. Although, their study used mannequins with fundus images as opposed to real eyes, the outcomes indicate the positive potential of smartphone ophthalmoscopy for clinical work. Work by Wu et al. (2018) found 92% of medical students included in their study (total *n* = 25) preferred the D-EYE to the direct ophthalmoscope [25]. The findings are somewhat in contrast to our results with optometry students who, despite a positive response to the D-EYE, preferred the direct ophthalmoscope to the D-EYE when assuming the role of a practitioner. The difference between optometry and medical students may lie in the amount of time spent undertaking direct ophthalmoscopy as part of their respective degrees. In the UK, optometry students typically learn direct ophthalmoscopy early in the degree process and continue to develop and refine their skills throughout the training period (which typically spans across 3–4 years). UK medical students, however, spend comparatively less time on ophthalmology and have been reported to spend an average of just 7.6 days on ophthalmology placements training during their degrees [27].

One of the challenges we, and presumably other educators, have experienced when teaching direct ophthalmoscopy are the limitations which result from a singular eyepiece and inability to demonstrate or record aspects of the examination. As educators, we felt a clear benefit of the D-EYE was the potential to examine a patient/volunteer while being able to describe and discuss retinal structures where both teacher and student could view the image simultaneously. This type of context specific instructional scaffolding approach may help students use the D-EYE as a precursor to direct ophthalmoscopy. The large image also allows and supports an opportunity for group learning and the possibility of peer-to-peer learning. Within a training context, the video recording element has potential for teaching and reflection purposes. In a clinical context, there is potential for D-EYE to be used for electronic record keeping and in telehealth.

In summary, while the D-EYE in its current form is not a replacement for the traditional direct ophthalmoscope, we believe it has a useful purpose in an educational context. The experiential element of learning combined with the context specific, situated learning [28, 29], as a group could help develop student confidence and precede the use of more technically challenging approaches to fundus examination. If the D-EYE technique develops an improved visualization of the undilated fundus beyond the central retinal (i.e. optic disc and macular) area, allowing for validation against the direct ophthalmoscope, then there may be potential to expand its use within clinical and educational settings.
